# Quantitative and textural analysis of magnetization transfer and diffusion images in the early detection of brain metastases

**DOI:** 10.1002/mrm.26257

**Published:** 2016-06-09

**Authors:** Nicola L. Ainsworth, Mary A. McLean, Dominick J.O. McIntyre, Davina J. Honess, Anna M. Brown, Susan V Harden, John R. Griffiths

**Affiliations:** ^1^ Cancer Research UK Cambridge InstituteUniversity of CambridgeLi Ka Shing CentreRobinson WayCambridgeCB2 0RE; ^2^ Department of OncologyAddenbrooke's HospitalHills RoadCambridgeCB2 0QQ

**Keywords:** MRI, cancer, magnetization transfer, diffusion, brain metastasis, texture analysis

## Abstract

**Purpose:**

The sensitivity of the magnetization transfer ratio (MTR) and apparent diffusion coefficient (ADC) for early detection of brain metastases was investigated in mice and humans.

**Methods:**

Mice underwent MRI twice weekly for up to 31 d following intracardiac injection of the brain‐homing breast cancer cell line MDA‐MB231‐BR. Patients with small cell lung cancer underwent quarterly MRI for 1 year. MTR and ADC were measured in regions of metastasis and matched contralateral tissue at the final time point and in registered regions at earlier time points. Texture analysis and linear discriminant analysis were performed to detect metastasis‐containing slices.

**Results:**

Compared with contralateral tissue, mouse metastases had significantly lower MTR and higher ADC at the final time point. Some lesions were visible at earlier time points on the MTR and ADC maps: 24% of these were not visible on corresponding T_2_‐weighted images. Texture analysis using the MTR maps showed 100% specificity and 98% sensitivity for metastasis at the final time point, with 77% sensitivity 2–4 d earlier and 46% 5–8 d earlier. Only 2 of 16 patients developed metastases, and their penultimate scans were normal.

**Conclusions:**

Some brain metastases may be detected earlier on MTR than conventional T_2_; however, the small gain is unlikely to justify “predictive” MRI. Magn Reson Med 77:1987–1995, 2017. © 2016 The Authors Magnetic Resonance in Medicine published by Wiley Periodicals, Inc. on behalf of International Society for Magnetic Resonance in Medicine. This is an open access article under the terms of the Creative Commons Attribution License, which permits use, distribution and reproduction in any medium, provided the original work is properly cited.

## INTRODUCTION

Brain metastases (BM) develop in 20 to 40% of all cancer patients and are a major cause of morbidity and mortality^1^. Neurosurgery or stereotactic radiosurgery may be possible in selected patients, but for the majority, treatment options include steroids, whole brain radiotherapy, and supportive care with median survival measured in a small number of months. Cumulative incidence of brain metastases is noted to be particularly high for patients with locally advanced lung cancer and HER2 positive metastatic breast cancer, with figures of up to 30–60% reported 2, 3, 4, 5. The high incidence of BM is perhaps the result in part of the use of targeted drugs such as monoclonal antibodies: These are effective in controlling extracranial metastatic disease and thereby extending survival, but they do not cross the blood‐brain barrier and are therefore ineffective against BM.

In light of the very high incidence of BM for patients with small cell lung cancer (SCLC), prophylactic cranial irradiation (PCI) was introduced into the routine management of patients with limited stage 2 and extensive stage 3 disease, and it significantly improves overall survival in addition to significantly reducing the incidence of BM. There have also been a number of trials investigating the role of PCI for non‐small‐cell lung cancer (NSCLC) 4, which show significant reduction in the incidence of BM but no significant difference in overall survival. Although one recent small study of PCI for breast cancer 6 hinted that PCI could reduce BM incidence at 2 years, it was underpowered for statistical significance.

Although PCI is routinely used in patients with SCLC, careful assessment of cognitive function does show a decline in memory and recall with PCI in survivors 7, and hippocampal sparing PCI may be helpful. Alternative strategies for patients at high risk for developing brain metastases from other primary sites, where PCI does not improve overall survival, include careful follow‐up with MRI to detect BM before they are symptomatic and when they might be most suitable for targeted treatment.

MRI is the gold standard for detection of brain metastases, but it is not universally given to cancer patients who lack neurological symptoms at presentation. Even in patients with a normal MRI brain scan at diagnosis and limited‐stage SCLC, a recent study found that 32.5% subsequently developed MRI evidence of brain metastases by the end of chemotherapy, before receiving PCI 5. If we could noninvasively identify any alteration in the brain microenvironment that precedes the current clinical detection of metastases, using techniques such as magnetization transfer ratio (MTR) or apparent diffusion coefficient (ADC), it may be possible to better individualize treatment regimes and improve survival.

The MTR of primary brain tumors has been shown to be reduced relative to normal brain in preclinical 8, 9, 10 and clinical studies 11, 12. In rat glioma models, the MTR has been shown to fall further with the development of the tumor 9. A small clinical study has shown a reduction in MTR both within metastases and outside of the contrast‐enhanced region 13. Most of the clinical brain metastases have increased ADC in comparison with normal brain; however, reductions have also been reported in highly cellular metastases such as in SCLC 14, 15.

Texture analysis (TA) is a method of mathematical classification of images based on the intensity variations within them. A number of features can be calculated from an image, based for example on spatial factors such as the local gradient of intensity changes or co‐occurrence of similar intensities within a certain distance 16. Histogram‐based features from the entire image may also be considered, as may higher‐order features such as spatial frequency distributions following wavelet transformations or fits to an autoregressive model 17.

Texture analysis has been evaluated as an automated tool for classifying brain tumor types and grades: It has been shown to have 100% accuracy in classifying benign from malignant lesions and up to 94% accuracy in discrimination of primary from secondary tumors 18. It has also shown utility, when compared with qualitative inspection of MRI, in automatic detection of more subtle cortical abnormalities such as focal cortical dysplasia, a malformation of cortical development that causes intractable epilepsy 19. Although usually applied to conventional MR images, it has also been applied to MTR images to identify patients with multiple sclerosis 20, and to ADC images to identify mild traumatic brain injury 21.

The aim of this study was to explore the potential of quantitative and textural analysis of serial MR imaging to detect and predict the development of brain metastases. The study was primarily carried out in mice using a brain‐homing breast cancer cell line, but a small pilot study in humans with SCLC was also performed in parallel.

## METHODS

### Animal Model

All animal experimentation was performed in accordance with the UK Animals (Scientific Procedures) Act of 1986 and with local ethical approval under PPL 80/2203. The brain‐homing breast cancer cell line MDA‐MB231‐BR (supplied by Prof. Joseph Frank at NIH laboratories, Bethesda Maryland, with permission from Dr. Diane Palmeri at NIH) was delivered via intracardiac injection into groups of no more than 10 female 6–7‐week‐old SCID mice from Charles River Laboratories (inoculum of 10^5^ cells in 100 µl). Typical body weight was 15–19 g, and remained static in mice developing metastases, while the maximum weight gain seen in control (saline‐injected) mice was 2 g. The final health end point was defined as the occurrence of limb weakness, seizures, or 20% weight loss.

Mice underwent MRI twice weekly from 14 d post‐intracardiac injection until the health end point previously defined; available magnet time typically allowed for scanning on days 14 or 15, 17 or 18, 21 or 22, and 24 or 25 and some later times. The mean time from injection to culling (the “final time point”) was 24 d (range 21–31; Table 1). Eight control mice injected with DPBS were also scanned on multiple occasions using the same scan protocol, with the final time point at day 21–24 and the early time point at day 18–21. Mice that underwent intracardiac injection of MDA‐MB231‐BR eGFP cells, but did not develop metastases visible on the MR images or reach the defined health end point, were kept and imaged for a further 2–3 weeks. On completion of imaging, or earlier if the animal became unwell, it was humanely killed by perfusion fixation under terminal inhalation anesthesia (2–4% isoflurane in 100% oxygen) and the fixed brain was paraffin‐embedded for histological examination.

**Table 1:** Summary of Mouse Scanning Schedule.

Interval (d)	Injection to final scan	Early to final scan	Very early to final scan
Mean ± SD	24.5 ± 3.7	3.0 ± 0.6	6.0 ± 1.2
Range	21–32	2–4	5–8
N (mice)	12	11	10

### Preclinical MRI

Mice were positioned head first, prone in a 72‐mm volume transmit coil with a quadrature brain receive coil (Rapid Biomedical, Würzburg, Germany) inside a 9.4 Tesla (T) Agilent MR scanner (Agilent Technologies, Yarnton, Oxford, UK) equipped with a gradient set of strength 40 G/cm, 120 µsec rise time and 120‐mm inner diameter. Motion was minimized using custom‐made padding, securing the limbs and tail, and using tooth and ear bars when feasible. Animals were anesthetized using inhaled isoflurane (Abbotts Laboratories, Berkshire, UK). Isoflurane was delivered in oxygen (1 L/min) at 2–3% for induction and 1.5–2% for maintenance as required. Excess gases were scavenged (Active Scavenging Unit, Vet Tech Solutions, Cheshire, UK). Core temperature and respiration rate were monitored using a Small Animal Instruments monitoring/control system (SA Instruments, New York, New York). The core body temperature was monitored using a rectal probe, and was maintained at 36.8 °C by warm air blown over the animal. Respiration rate was measured using a respiratory pillow taped to the mouse's abdomen and attached to a pressure transducer. The respiratory rate was recorded and maintained at 50–110 breaths per minute by adjustment of the level of anesthesia.

The brain was shimmed manually on the free induction decay (FID) to achieve a line width of <75 Hz. T_2_‐weighted fast spin echo localizers were acquired in the axial plane (echo time (TE)/pulse repetition (TR) = 48/2000 ms, 128 × 128, field of view (FOV) 20 mm, chemical shift selective (CHESS) fat saturation, 15 slices, 0.5‐mm thick, 0.2‐mm gap, interleaved, echo train length (ETL) 8, echo spacing 12 ms, 8 averages). Coronal slices were prescribed using anatomical landmarks on the central axial slice in matching locations for high‐resolution T_2_ (TE/TR = 35/2000 ms, 160 × 160, 25‐mm FOV, 15 interleaved slices, 0.3‐mm thick, 0.1 mm gap); diffusion‐weighted echo planar imaging (EPI) (TE/TR = 23.9/2000 ms, 24 shots, ETL 6, 4 averages, 128 × 128, 25‐mm FOV, 15 slices, thickness 0.3 mm, gap 0.1 mm, acquiring one image with minimal diffusion weighting (b = 22.4 s/mm^2^) and three with b = 489 s/mm^2^ and diffusion gradients oriented along each of the three orthogonal gradient axes); and magnetization transfer (three‐dimensional (3D) gradient echo with narrow‐band off resonance pulses with flip angles of 1350 ° and 400 ° and frequency offsets of 4000 and 80000 Hz, respectively, for magnetic transfer (MT) on/off, TR 30 ms, 10 ° flip angle, 128 × 128 × 32 points, 25 × 25 × 12.8 mm FOV, slab thickness 11 mm, 8 averages). All images were reconstructed to 256 × 256 resolution in plane, and trace (ADC) images were calculated using software provided by the manufacturer. The MRI protocol took 2–2.5 h per mouse, depending on the time taken to anesthetize and shim the mouse before scanning. We chose to use similar spatial resolutions for each sequence, so that partial volume effects with small metastases would be similar. With this constraint, signal‐to‐noise ratio (SNR) and restrictions on anesthesia duration prevented the use of enough gradient directions for a full diffusion tensor imaging experiment; we instead acquired three orthogonal directions, enough to compute the trace of the diffusion tensor.

### Preclinical MRI Analysis

Preclinical MR images were analyzed using ImageJ (v1.5; National Institutes of Health, Bethesda, Maryland) and locally developed plugins. MTR images were calculated after thresholding according to

(1) $$MTR=100\times \frac{M_{0}-M_{s}}{M_{0}}$$

where M_0_ is the image with the low‐power high‐offset saturation pulse, and the M_s_ is the image with the high‐power low‐offset pulse.

Brain extraction was performed on the T_2_‐weighted images using a freely available 3D pulse‐coupled neural network (PCNN) program 22, 23 implemented in MATLAB (Math Works, Natick, Massachusetts) with in‐house modification and a manually selected cutoff for edge delineation. The brain mask was then applied to the diffusion‐weighted images (DWI) and MTR images in MATLAB.

The skull‐stripped 3D volumes from each of the early time points of the T_2_‐weighted images were independently registered to the final time‐point images using ANTS (Advanced Neuroimaging Tools) 24, which uses a linear transform derived from a mutual information similarity metric. The affine and warp transforms calculated were applied to both the T_2_‐weighted and ADC images from early time points to spatially align them with the final time point. Because the MTR images have inverted contrast relative to the T_2_‐weighted images, they were registered separately following the same method.

**Figure 1:**
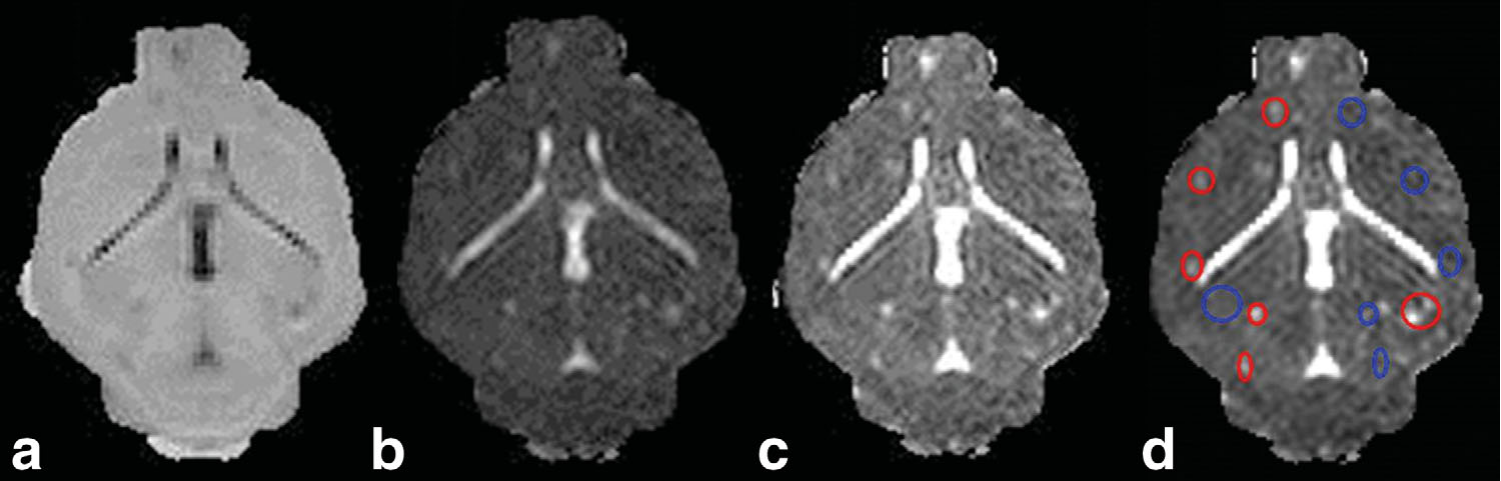
Example of ROI definition in mouse MRI at the final time point. MTR map (a), ADC map (b), map of ADC divided by MTR, to maximize conspicuity of lesions (c), ROIs defined (metastases are shown in red and matched contralateral brain in blue) (d).

ROIs were drawn in ImageJ around metastases at the final time point and at matched contralateral locations. To maximize lesion visibility, ROIs were drawn on images of ADC divided by MTR, then applied to the individual parameter maps of MTR and ADC (Fig. 1). Descriptive statistics for each region of interest (ROI) (mean, standard deviation (SD), median, and diameter) were recorded for the MTR and ADC images at the final time point and at all earlier time points, which had been registered as described previously. ROI analysis results were analyzed using Microsoft Excel (Microsoft, Reading, UK) and GraphPad Prism (version 6.00 for Windows, GraphPad Software, La Jolla, California).

### Texture Analysis

The maps of MTR, ADC, and ADC/MTR were converted to 16‐bit integer data, scaled such that the full integer range corresponded to the range 0–1 in the original MTR map and ADC/MTR images, and 0–0.004 for ADC. Slices 7 to 13 from the top of the standard 15‐slice data set were analyzed, as these slices all contained metastases in the metastasis‐positive mice. Control data consisted of the same slices from the sham‐injected mice. Any apparent imperfections in the skull‐stripped brain masks were corrected using a combination of thresholding and erosion in ImageJ, followed by manual dilation of the bitmask in MaZda version 4.6 (Institute of Electronics, Technical University of Lodz, Poland) 17.

In MaZda, the image intensities were normalized to the mean ±3 SD, and the full set of over 300 texture parameter values were calculated for each slice. The top 30 features that differed most significantly between metastasis and control slices acquired at the final time point (training set) were selected using the Fisher, minimization of classification error probability and average correlation coefficients (POE+ACC), and mutual information (MI) methods within MaZda. Following this feature reduction, linear discriminant analysis (LDA) was performed using b11, the statistical package within MaZda, on the images from metastasis and control mice to optimize the separation of the classes, as visualized by plotting the MDF‐1 (the first most discriminating feature) 25 in GraphPad Prism.

MTR maps from both control and metastasis‐positive mice acquired at earlier time points (the penultimate time point, “early metastases” and “early control”, and for metastasis‐positive mice at the time point before, “very early metastases”) were also evaluated using texture analysis (the test sets). The MDF‐1 was calculated using the same top 30 features, weightings, and formulas optimized for the training group.

### Clinical MRI

The study protocol was approved by the Cambridge Research Ethics Committee and all subjects provided written, informed consent. Patients with a histological diagnosis of SCLC with no contraindications for MRI were studied on four occasions (at diagnosis, post‐chemotherapy at approximately 4 months, post‐PCI at 8 months, and at 1 year) on a 3T HDx scanner using body coil transmission and an eight‐channel phased‐array head coil (GE Healthcare, Waukesha Wisconsin). The protocol included the following, all acquired in the axial plane: T_2_‐weighted fast spin echo (TE/TR = 98.7/7000 ms, ETL 32, FOV 22 cm, matrix 512 × 384, ASSET acceleration factor 2, slice thickness of 5 mm with 1‐mm gap), fluid‐attenuated inversion recovery (FLAIR) (TE/TI/TR = 121.7/2000/7000 ms, FOV 22 cm, matrix 384 × 224, slice thickness 5 mm, 1‐mm gap), single‐shot EPI spin echo diffusion tensor imaging (23 slices, 5‐mm thick, 1‐mm gap, ASSET factor 2, TE/TR = 76.8/6000 ms, FOV 22 cm, matrix 128 × 128, b values of 0 and 1000 s/mm^2^ along 25 gradient directions), magnetization transfer using a 3D fast spoiled gradient echo (FSPGR) (TE/TR = 1.1/28.5 ms, FOV 22 cm, matrix 256 × 160, 60 × 3 mm slices, 8 ms Fermi MT pulse with frequency offset 2200 Hz and a nominal flip angle of 450 °), pre‐ and postgadolinium T_1_‐weighted FSPGR (TE/TI/TR = 3.9/450/9.5 ms, flip angle 20^o^, bandwidth 31.25 kHz, FOV 22 cm, matrix 352 × 224, ASSET factor 2, 120 × 1.2 mm slices, Gadopentetate dimeglumine (Gd‐DTPA) delivered via an intravenous bolus of 0.1 mmol/kg). Maps of ADC and FA were computed by the scanner software, and MTR maps were computed offline using scripts in FSL (FMRIB Lab, Oxford, UK).

## RESULTS

### Preclinical MRI

Of the 29 mice injected with MDA‐MB231‐BR cells and scanned on multiple occasions, 14 developed brain metastases (48%). Four of these did not have diffusion data collected as a result of technical problems early in the study. For the 10 mice with the full MRI protocol acquired, the mean number of brain metastases observed was 32 (range 13–53). Because the metastases often spanned multiple slices, the average number of ROIs drawn was 44 (range 13–158), with average diameter 0.8 mm (range 0.5–1.7).

**Figure 2:**
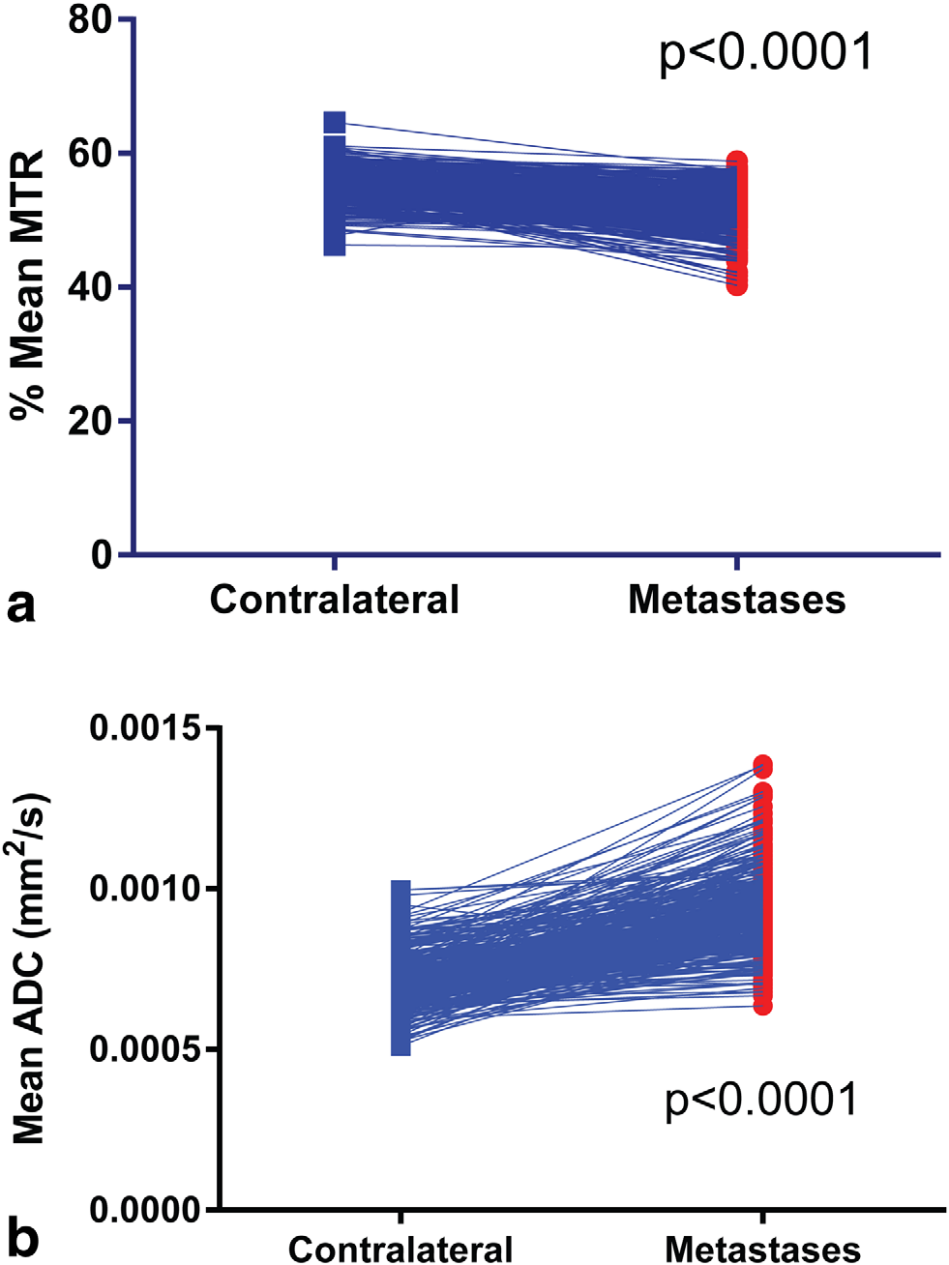
Paired values of MTR (a) and ADC (b) in metastasis and contralateral ROIs. For each, *P* < 0.0001 in paired t‐tests.

**Figure 3:**
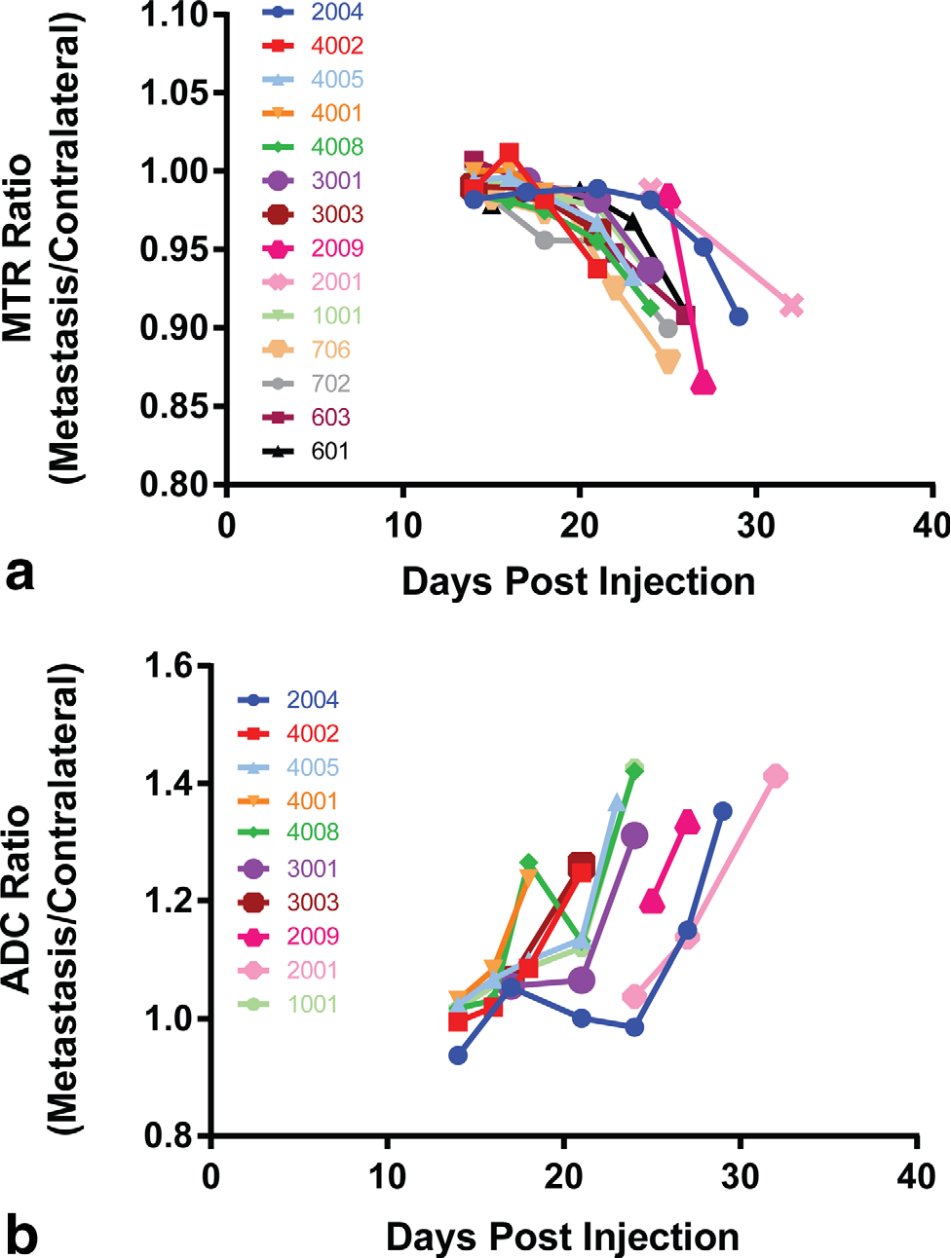
Time course of the ratios of values in metastases divided by those in the matched contralateral ROIs for MTR (a) and ADC (b). All time points were registered to the final images acquired for that animal prior to culling at day 21–32.

**Figure 4:**
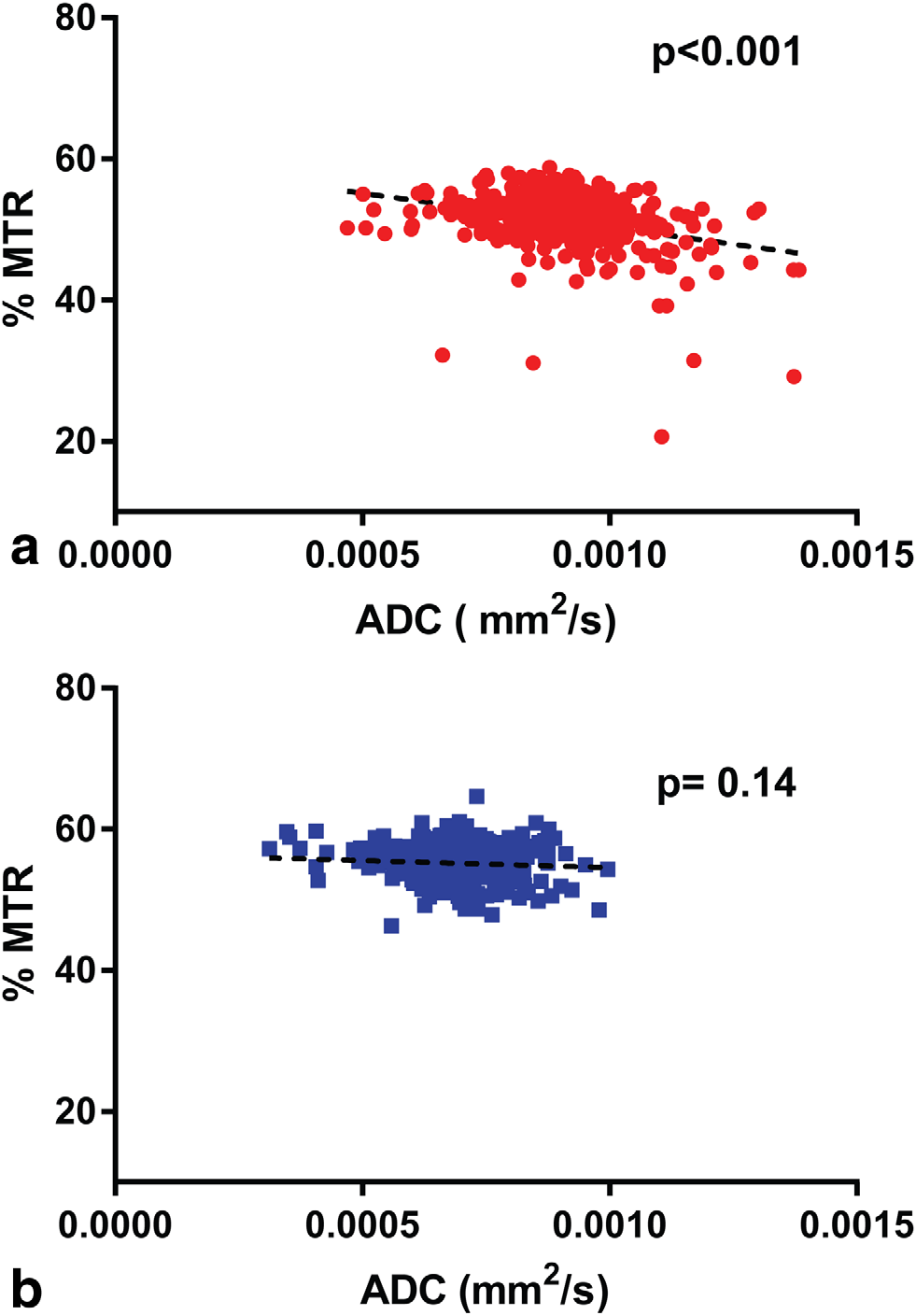
Correlation between the mean MTR and the mean ADC in ROIs drawn in metastases (top, red) and contralateral brain (bottom, blue). A significant negative correlation is found when there is a metastasis present, but not in normal‐appearing brain.

Quantitative analysis of MTR and ADC at the final time point showed significant differences on paired t‐tests between the metastases and ROIs at matched contralateral locations (Table 2; Fig. 2): The metastases had lower MTR and higher ADC (*P* < 0.0001 for both). The use of paired ROIs is helpful, as the parameters can vary in different parts of the brain. MTR in particular is known to be higher in white matter than gray 26. The ratio of metastasis to contralateral regions was plotted for each parameter measured in serially registered images from the same mice over time (Fig. 3), showing that although there was variation in the lag before changes were measurable, once initiated, the rate of decrease in MTR and increase in ADC was consistent among animals. There was a significant negative correlation between MTR and ADC in metastasis voxels but not in contralateral normal‐appearing brain (Fig. 4).

**Table 2:** Summary Statistics for Parameters Measured in ROIs Drawn in Metastases and Matching Contralateral Locations in Mouse Brain at the Final MRI Time Point Prior to Culling.

	MTR (%)	ADC (×10^−3^ mm^2^/s)
Metastasis	51.4 ± 3.2	0.93 ± 0.13
Contralateral	55.3 ± 2.5	0.71 ± 0.08
Paired difference	3.8 ± 2.9	0.22 ± 0.13
t‐statistic	25.9	32.8
N	381	357

**Figure 5:**
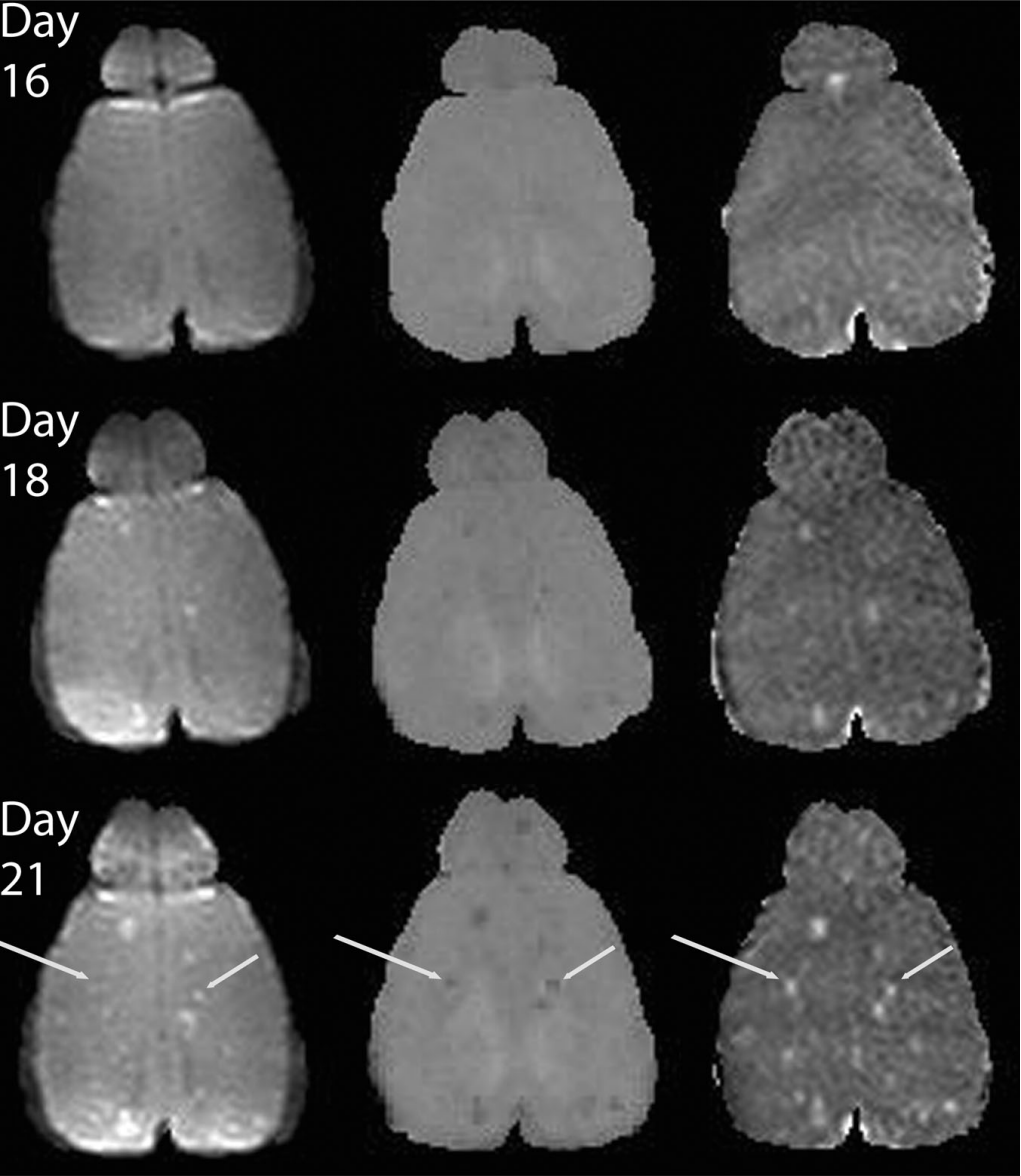
T_2_‐weighted (left), MTR (middle), and ADC (right) images from the same mouse at three different time points (days 16, 18, and 21). Some lesions (long arrow) can be seen on the MTR and ADC image but not the T_2_‐weighted images.

Metastases were seen in most mice at approximately day 21, with some detectable at day 18. All of the metastases visible on the T_2_‐weighted images were also identified on the MTR and/or the ADC images; however, in 24% of the cases, metastases could be seen on the MTR and ADC images but not on the T_2_‐weighted ones. For example, Figure 5 shows a metastasis that was visible on ADC and MTR images but not on the T_2_‐weighted image (long arrows). This same metastasis can be seen in the MTR and ADC images at day 18 as well. The diameter of this metastasis (measured in ImageJ on the MTR image) was 0.37 mm, larger than the metastasis highlighted by the short arrow, which is clearly seen on all images (0.30 mm), so the invisibility on T_2_ is not solely due to size—particularly because the T_2‐_weighted images were acquired at the highest spatial resolution (in‐plane pixel diameter 0.16 mm, versus 0.20 for DWI and MTR).

### Texture Analysis

**Figure 6:**
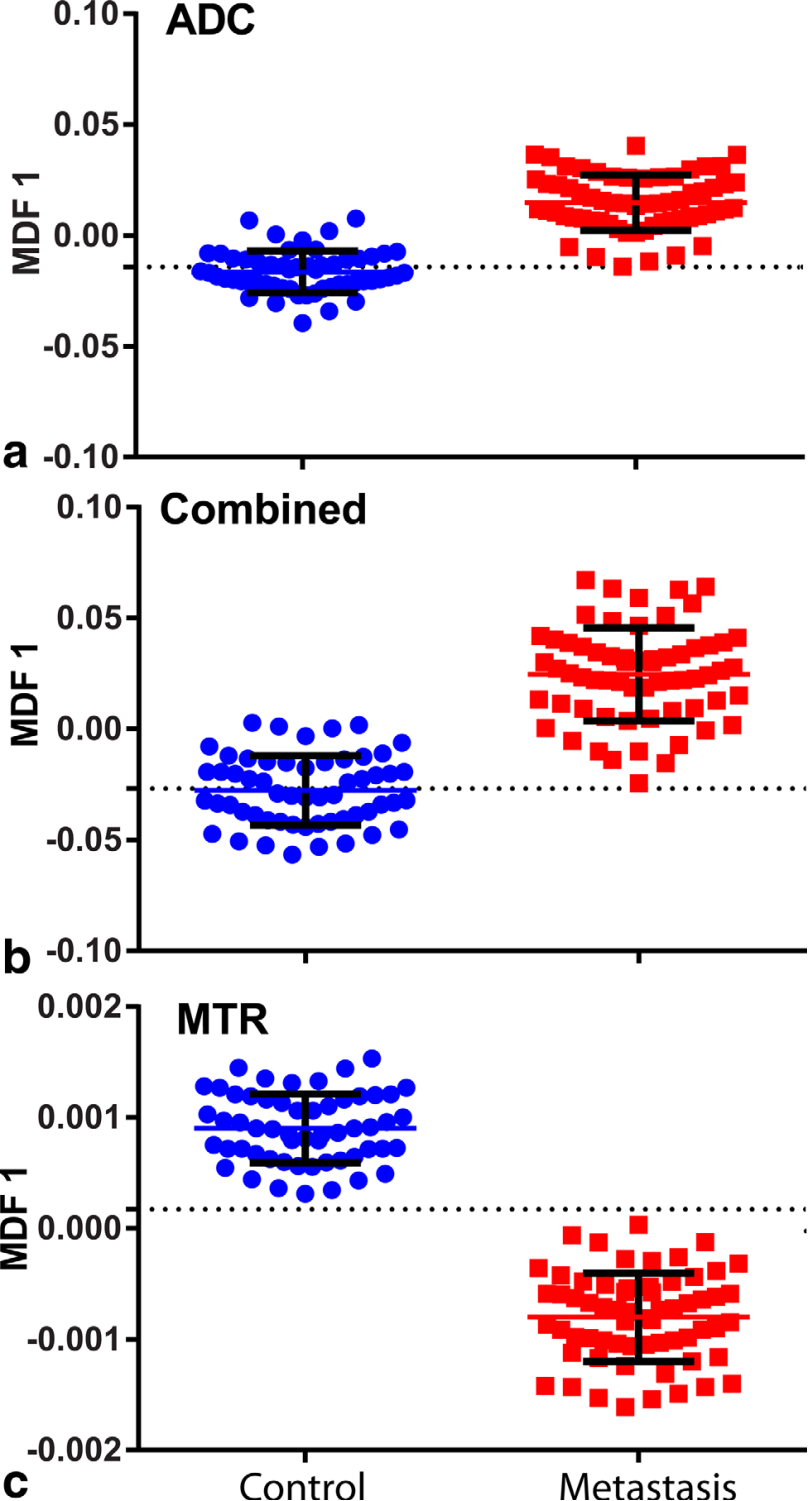
Most discriminating feature (MDF‐1) for classification of image slices from the final time point as control (blue) versus metastasis (red) based on texture analysis, for ADC (a), the combined ratio ADC/MTR (b), and MTR (c). The dotted line is drawn at the point of 100% sensitivity for detecting metastases.

Comparison of the MRI scans with histology showed that MRI underestimated the size of visible lesions and failed to detect many smaller lesions (data not shown). Therefore, we investigated whether the sensitivity to abnormality could be enhanced by performing texture analysis, examining separately the maps of MTR, ADC, and the ratio ADC/MTR. The best texture analysis classification of final time‐point slices into metastasis versus control was found when using MTR data alone (Fig. 6; 100% specificity, 98% sensitivity).

**Figure 7:**
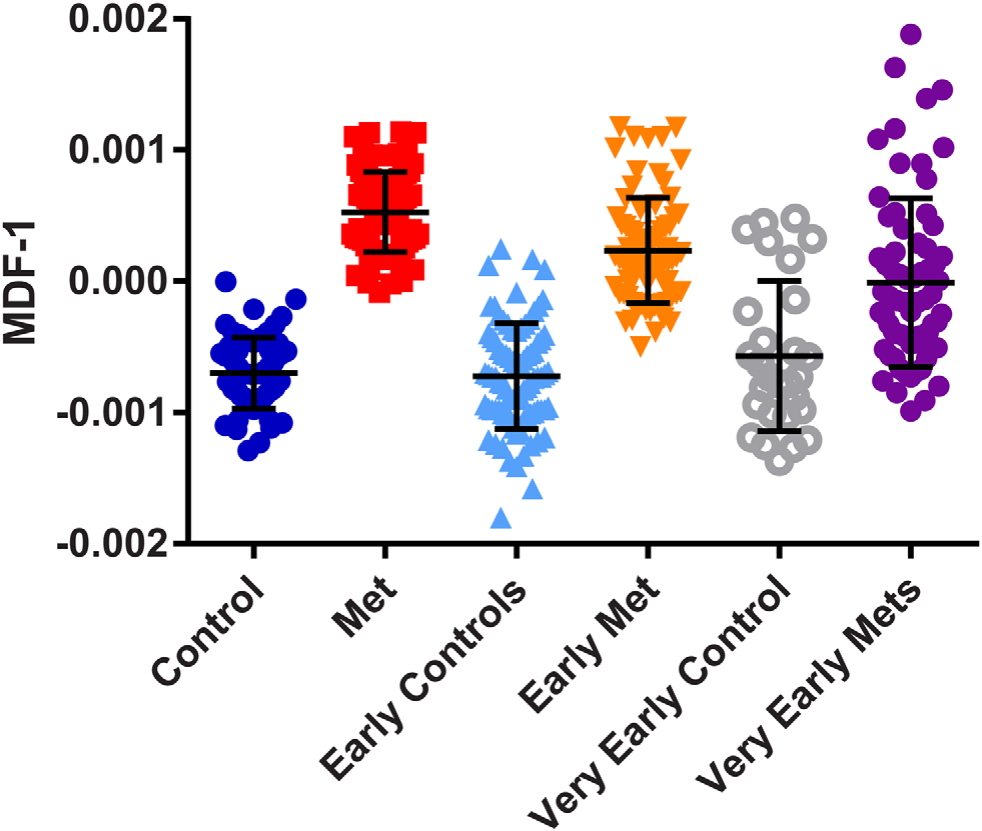
Classification of early and very early time‐point data as metastasis versus control using texture analysis of MTR maps. Bars indicate the mean ± 1 SD of the MDF‐1 value for each group, and the dotted line indicates MDF‐1 = −−0.0001112, the 100% specificity threshold determined on the training set from the final time point (left two columns, also shown in Fig. 6). The MDF‐1 value correctly classifies 58 of 63 slices from early controls and 59 of 77 slices from early metastasis, but only 22 of 28 from very early controls and 32 of 70 slices from very early metastasis.

We then examined whether slices from the same mice at earlier time points could also be classified as metastasis or control using the MDF‐1 calculated from the MTR map texture parameters with the same formula as the final time‐point training set. At the early time point (2–4 d before culling), the sensitivity for identifying an image slice as containing metastases was 77%, with 92% specificity (Fig. 7). At the very early time point (5–8 d before culling), sensitivity was only 46% and specificity 79%. Taking the mean MDF‐1 over all slices for each mouse, 9 of 11 (82%) would be classified as abnormal at the early time point, and 4 of 10 (40%) at the very early time point.

### Patient Details

Sixteen patients consented to the CLUB‐01 trial: The mean subject age was 65 (range 51–78 years), and 63% were male. None were found to have metastases at diagnosis, and only two (12.5%) developed brain metastases over the course of the study, considerably fewer than the incidence reported in other trials 2, 4, 27, 28. One of these (Patient 9) had asymptomatic metastases detected at the final scan (four lesions, mean diameter 5.4 mm, range 3.4–7 mm), whereas the other (Patient 10) developed neurological symptoms after four cycles of chemotherapy and was found on CT to have six metastases (mean diameter 4.8 mm, range 2.3–7 mm). None of these metastases were associated with edema. The time interval between the final and penultimate scans was 4 months and 12 d for Patient 10 and 3 months and 10 d for Patient 9 (and contrast was refused at the penultimate scan for Patient 9).

### Patient MRI

**Figure 8:**
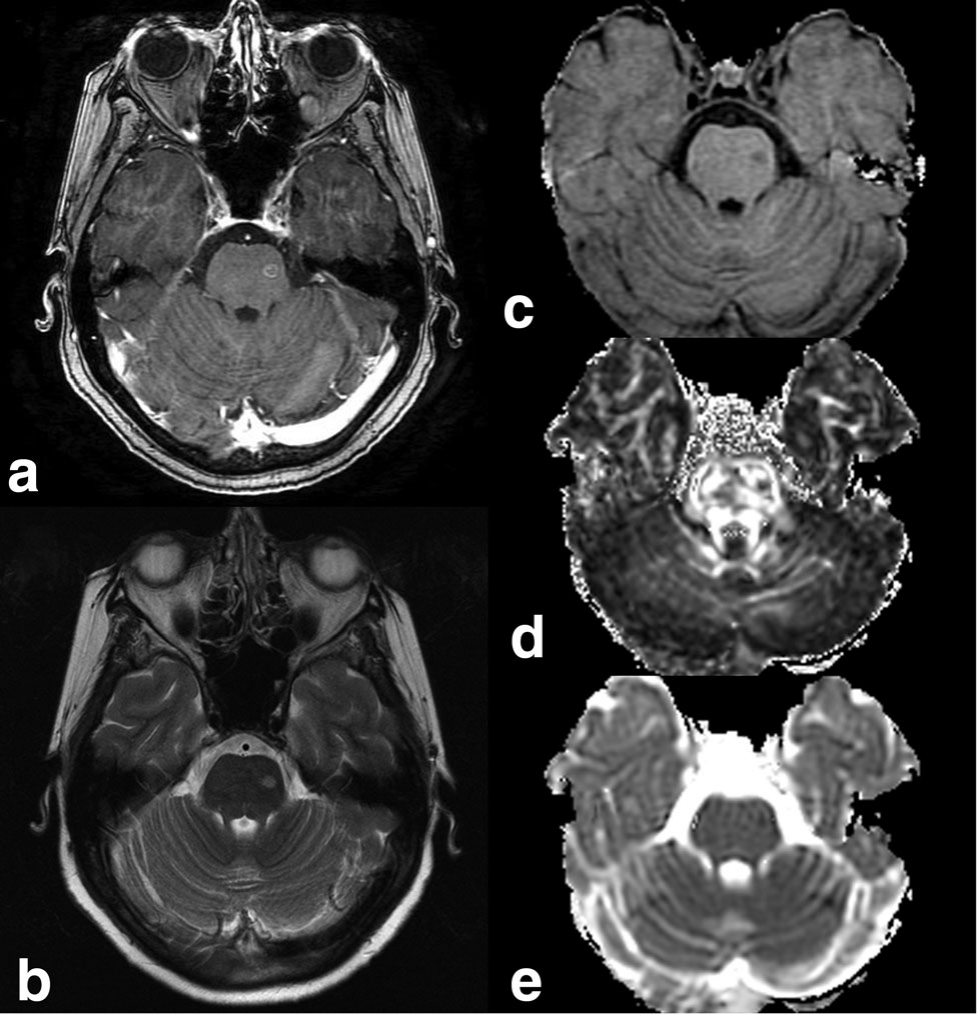
Anatomic images and parameter maps acquired following the development of neurological symptoms in Patient 10. Metastasis is seen in the brainstem in T_1_‐weighted images post‐Gd (a), T_2_‐weighted images (b), MTR maps (c), and FA maps (d), but not in ADC maps (e).

Because there were only two cases of metastasis development, no meaningful statistical comparisons could be performed; however, the imaging findings are of interest. Metastases were generally invisible on ADC maps but could be seen as areas of reduced signal on maps of MTR and FA at the final time point (Fig. 8). No abnormalities were detected on the penultimate scan (data not shown).

## DISCUSSION

The aim of this project was to optimize early detection of cerebral metastases using quantitative MTR and ADC imaging techniques, in combination with textural analysis (TA) and other image processing techniques. The strategy was biweekly sequential imaging of brains of mice injected with the MDA‐MB231‐BR cell line, before and after the development of visible metastases. Once the metastases were seen, we examined the same regions on earlier scans to see whether there were subtle changes that could be visualized using our methods (as a result of, for instance, the establishment of premetastatic niches 29).

We found, as expected, a fall in the metastasis to contralateral ratio of MTR and a rise in the corresponding ADC ratio (Fig. 3); however, these ratios did not differ significantly from 1.0 until the last 2–3 time points, when metastases began to be visible in the images. Although ultimately the magnitude of change in this ratio was greater for ADC than for MTR, the shape of the curves was more consistent for MTR, potentially allowing earlier identification of developing abnormality. For ADC, apparent early increases were often followed by levelling off or decreases, indicative of variability in the pattern of development and/or of imprecision in the measurement procedure.

There was a significant negative correlation between MTR and ADC within ROIs drawn in lesions, but not in normal‐appearing brain (Fig. 4). In a rat metastasis model using the same cell line 30, tumors were found to have elevated ADC, which corresponded to the extensive edema seen on histology. The ADC increased further as the tumors grew. Magnetization transfer has not previously been studied in this model, but reduced MTR has been seen previously in rat glioma models 4, 8, 9, 31 as well as in clinical brain metastases 13. A recent study investigated the development of brain metastases induced by intracerebral injection of ENU1564 cells in rats, using multimodal MRI. In keeping with our data, they found that the MTR was the most sensitive measure of micrometastatic growth, with a reduction in MTR evident as early as 7 d postinjection 32.

The edema seen in this model, associated with an increase in the free water content, would be expected to decrease MTR as well as to increase water diffusion. Additionally, myelin is thought to be a major contributor both to the MT effect 33, 34 and to the restriction of water diffusion across axons 35, so the displacement or replacement of myelinated axons by proliferating tumor cells would be expected also to decrease MTR and increase ADC in parallel.

In some cases, the changes in MTR and/or ADC preceded the appearance of a detectable metastasis on T_2_‐weighted imaging (Fig. 5). However, in the mouse model, this increased the window of detectability by at most a few days, and only for a subset of lesions (approximately 24%). We did not systematically evaluate lesion detectability using Gd contrast agents, as our preliminary experiments confirmed the findings of other groups that blood brain barrier permeability is limited in these small metastases, which co‐opt host blood vessels with an intact blood‐brain barrier for their blood supply 30. Phase contrast imaging has been suggested to perhaps be more sensitive than dynamic contrast enhanced (DCE) MRI as an early indicator of vascular remodeling during metastasis development 36.

Texture analysis of mouse brain MTR maps was able to classify images as metastatic or control, both on a training set of final time‐point data and on a test set of images from 2–4 d earlier. Although TA could detect metastases when they were small and asymptomatic, it was unable to reliably recognize any subtle alterations in the brain preceding the appearance of metastases on the images.

Early metastases in patients showed reductions in MTR similar to the animal model; however, ADC did not increase, perhaps as a result of the lack of edema or macroscopic necrosis associated with these small metastases (Fig. 8). Studies correlating ADC with histology have demonstrated that the ADC increased in areas of necrosis but not in areas of viable tumor in mice with squamous cell tumors 37, in rat gliomas 8, and in clinical brain metastases 38. The lack of change in the ADC in small tumors in the CLUB‐01 patients suggests that they had not yet outgrown their blood supply sufficiently to become necrotic. The degree of cellularity and differentiation may also affect ADC values: highly cellular and poorly differentiated tumors have been found to have a lower ADC then less cellular and better differentiated tumors 14, 15. Brain metastases from patients with lung cancer may have particularly low ADC: Hayashida et al 15 reported a lower ADC in SCLC brain metastases (1.19 × 10^−3^ mm^2^/s) than even poorly differentiated adenocarcinomas (1.40 × 10^−3^ mm^2^/s).

Although the changes in ADC were more equivocal in humans than in mice, reductions in FA were striking. We were unable to measure FA in the mouse model because of SNR and examination time limitations, but it seems likely that FA and MTR would be sensitive to early metastasis development, because they are high (in the case of MTR, uniformly high) in normal white matter, and are markedly reduced in metastases regardless of the presence or absence of edema. Gaps in normal white matter may perhaps be detectable earlier than the presence of abnormal cancerous cells using these techniques.

We were unable to detect any abnormalities on the penultimate scan in the patients studied. The interscan interval of over 3 months was long, but more frequent scanning would be impracticable: if a predictive MRI test were to be useful, it would need to detect metastases that would first appear on conventional imaging a few months after the screening date.

More significant improvements in early detection of metastases have been shown using cell labeling: microparticles of iron oxide (MPIOs) allow direct monitoring with MRI of single cells when injected into preclinical models. The labeled cells are seen as hypo‐intense voxels on T_2‐_weighted or $T_{2}^{*}$‐weighted images. Heyn et al 39 have used this model to evaluate the formation of brain metastases from single MDA‐MB231‐BR cells at 1.5 T. Song et al 40 used a rat model to evaluate the MDA‐MB231‐BR cell line transfected with luciferase, and labeled with ferumoxides‐protamine sulphate (FEPro). Using $T_{2}^{*}$‐weighted MRI imaging, they could identify micrometastatic tumors 2 weeks after injection. In clinical studies it is not possible to label the tumor cells directly with MPIOs to allow easier MRI evaluation. Another approach, which could be applied in the clinical setting, is to conjugate the MPIO to targeted ligands, allowing the MPIOs to accumulate in areas where specific molecules are expressed. MPIOs have been conjugated to anti‐VCAM1 antibodies (vascular cell adhesion molecule 1), which were injected into mice that had also received intracardiac injection of MDA‐MB231‐BR cells. This process allowed MRI visualization of any up‐regulation of VCAM expression related to the brain metastases 41. Early up‐regulation of VCAM1 was found in vessels related to micrometastases, with expression increasing with tumor growth. This up‐regulation allowed detection of the metastases 5 d after injection. The authors discuss the translation to clinical studies; however, the currently available MPIOs are nondegradable, and therefore not suitable for use in patient studies.

## CONCLUSIONS

This study shows some evidence that a subset of brain metastases may be visualized on MTR and DWI earlier than on T_2_‐weighted imaging. The small expansion of the window of detectability, albeit in a rapidly growing model system, is unlikely to justify “predictive” MRI scanning. However, screening with brain MRI at diagnosis might be useful in management of small‐cell lung cancer, and this is not yet current practice across Europe.
